# Informatics derived materials databases for multifunctional properties

**DOI:** 10.1088/1468-6996/16/1/013501

**Published:** 2015-01-13

**Authors:** Scott Broderick, Krishna Rajan

**Affiliations:** Institute for Combinatorial Discovery and Department of Materials Science and Engineering, Iowa State University, Ames, IA 50011, USA

**Keywords:** materials informatics, scintillator, quantitative structure–property relationships

## Abstract

In this review, we provide an overview of the development of quantitative structure–property relationships incorporating the impact of data uncertainty from small, limited knowledge data sets from which we rapidly develop new and larger databases. Unlike traditional database development, this informatics based approach is concurrent with the identification and discovery of the key metrics controlling structure–property relationships; and even more importantly we are now in a position to build materials databases based on design ‘intent’ and not just design parameters. This permits for example to establish materials databases that can be used for targeted multifunctional properties and not just one characteristic at a time as is presently done. This review provides a summary of the computational logic of building such virtual databases and gives some examples in the field of complex inorganic solids for scintillator applications.

## 1. Introduction

Even with advances in high speed computing, there are limitations to the number of new material chemistries for which properties can be calculated. While electronic structure calculations provide a physical basis on which to propose new materials, the complexity of the calculations and the large amounts of data created cause a lack of design rules for rapidly designing new ‘virtual’ materials. To address these challenges, we have integrated statistical learning approaches with both computational and experimental data for a wide range of material classes. Developing quantitative structure–property relationships (QSPRs) for classes of materials provides an important paradigm for materials design by (i) utilizing the minimal amount of information needed for accurately modeling the property, thereby resulting in physically interpretable calculations; (ii) removing restrictions on the materials possible and requirement of pair-wise interactions; and (iii) enhancing the computational efficiency well beyond other physics based approaches.

High throughput electronic structure calculations have been previously developed and provide powerful and extensive computational strategies based on electronic structure calculations to search large virtual chemical spaces to identify potentially new compounds [1–5]. However, we demonstrate a different approach to establish such a structure–property relationship where we do not assume any specific formulation linking structure with property. Rather, we take a data-driven approach where we seek to establish structure–property relationships by identifying patterns of behavior between known discrete scalar descriptors associated with crystal and electronic structure and observed properties of the material. From this, we extract design rules that allow us to systematically identify critical structure–property relationships, resulting in identifying in a quantitative fashion the exact role of a specific combination of materials descriptors that govern a given property. Such formulations quantitatively define the impact of multi-scale data associated with electronic and crystal structure and materials properties.

QSPRs provide a high-speed screening of new chemistries. The purpose of this review is to develop a general template of the approach for computationally generating very rapidly large numbers of combinations of chemistry and microstructure, after which electronic structure calculations and experiments can be used to refine issues with the prediction. QSPRs define the relationship between the controllable characteristics of a material (particularly chemistry and processing) and properties. Beyond providing a high-speed modeling approach, QSPRs also provide meaningful relationships by defining the relationship between a few critical factors of the constituent material components and the overall material behavior. These models may be developed for multiple properties so that the material aspects which improve numerous and possibly inversely related properties can be identified, thereby accelerating targeted design well beyond what is possible via an Edisonian design strategy. This approach seeks to extract fundamental knowledge of materials behavior from a limited training data, which can be expanded without the requirements and assumptions of traditional materials design. Developing a model to link material characteristics and material properties accelerates the design of new materials, while also guiding the future computations and experiments needed by defining spaces of missing data.

In this review, we describe a standardized framework for development of QSPRs and the associated virtual material databases. We describe the approach for reducing the required descriptor space with rough set theory (RST) which incorporates uncertainty into the calculation [6–8], the development of QSPRs with multi-collinear regression approaches, and the development of virtual databases. In this review we will use inorganic scintillators as a case study as it provides a good example of the typical challenge of designing materials with multifunctional attributes, in this case a high light yield (LY) and fast decay times for applications in ionizing-radiation detectors [9–11]. We seek to identify the features of a material which contribute to the desirable properties and can therefore be used to accelerate scintillator design. This review builds on our prior works in developing QSPRs related with piezoelectrics, hard spinels, graphene nanoribbons, nanoparticles for drug delivery, and density of states spectra for new materials [12–18].

The following discussion first provides a background to the informatics techniques used for both classification and predicting new data and then provides the details of a test case of data in scintillators as a template that can be used for any future materials databases.

## 2. Computational strategy

The first objective of our approach for developing QSPRs which we then use to develop virtual materials databases is to minimize the assumptions of what is most important for a materials property. As opposed to a trial-and-error approach which is based on existing knowledge, our approach makes no assumptions as to what characteristics of a material dictate its properties. We instead utilize all existing data and then mathematically define the relationship between materials properties and the descriptor base. Our approach addresses challenges in database searches due to fuzzy classifications between material classes, such as between scintillators and phosphors, as well as the bias in the data since negative results are usually under reported. By addressing these issues, we build robust models when the data is skewed or sparse.

An important point to make about the informatics process is that the sole reliance on mathematics does not remove the known physical theory or intuition from the design process. That is, to have confidence in the design rules, particularly when dealing with sparse data or data with high uncertainty, a physical explanation for the rule is needed. The role of the QSPR strategy is therefore to make the explanation of governing physics more understandable by reducing the complexity of the problem to the minimal information needed. We then have the starting data and the final result, thereby making the intermediate physics more understandable than otherwise possible. This approach represents not just a data creation process but also an approach for understanding the driving physics.

**Figure 1.**
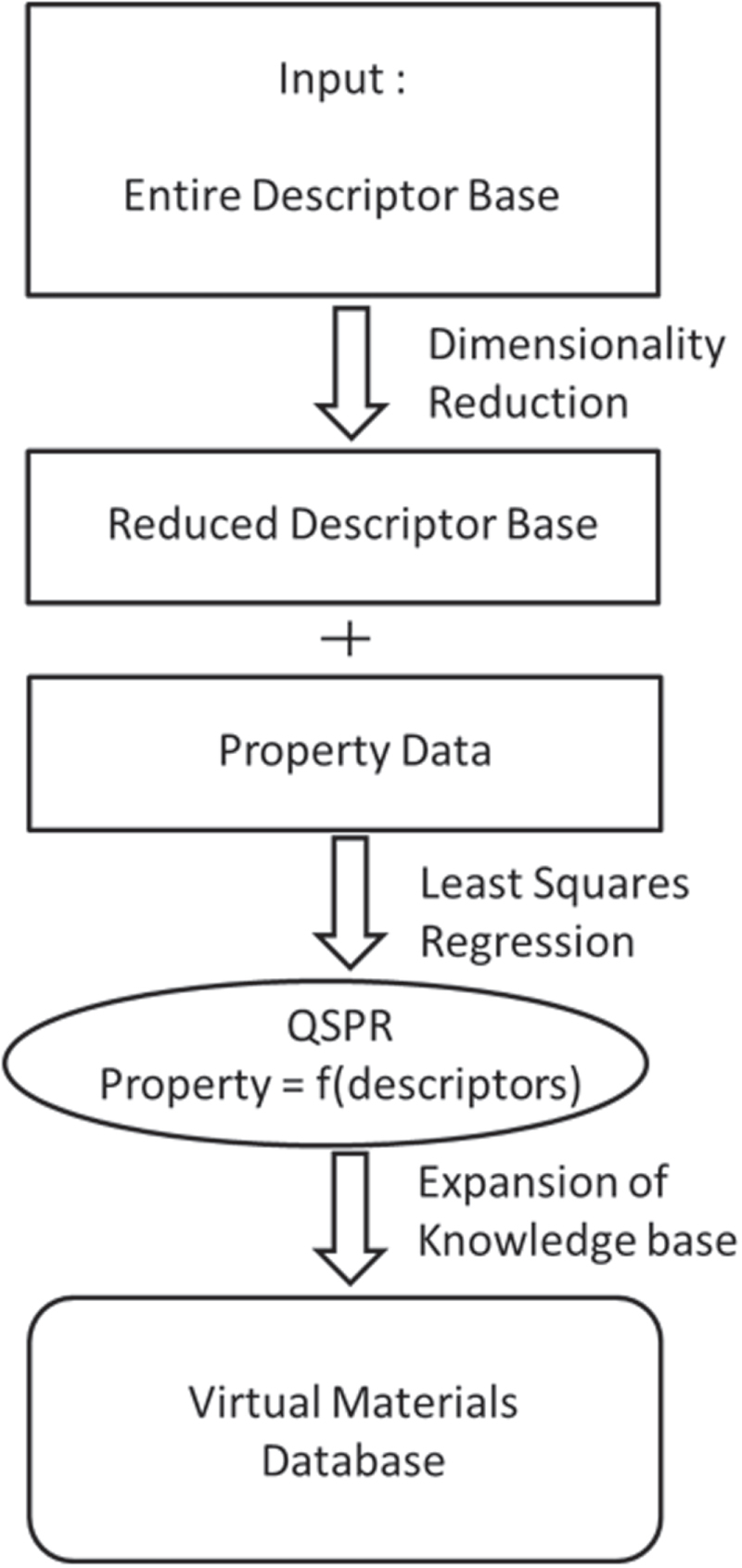
Standardized logic for developing virtual materials databases. This approach utilizes the entire material search space without *a priori* assumptions, identifies the minimum amount of information to describe the relevant problem, develop physics-driven design rules linking material and property, and then expansion of the materials knowledge base. In this review, we demonstrate the applicability of this approach to multiple material classes and problems.

The approach for defining QSPRs, which we have previously utilized for accelerating data creation, leads to a discovery process far beyond iterative design. This approach has several factors which address challenges with traditional design. First, we consider the entire descriptor base, reducing any initial assumptions as to what is important in a materials property. Instead, this approach makes no assumptions as to what data is most important and instead the descriptors dictating properties are defined in a totally unbiased manner. Applying dimensionality reduction, the descriptor base is minimized to only those descriptors providing information that is both not redundant and also relevant to the properties of interest. The advantage of reducing the descriptor base is two-fold. First, the data measurement requirements are significantly reduced by minimizing the necessary amount of starting data. Second, by minimizing the data, the accuracy of the predictions increases by reducing the likelihood of over-fitting the data. This reduced descriptor base is then compared with property data with a least squares approach. In this case, we utilize partial least squares (PLS), which is similar to a multivariate regression while addressing co-linearity in the data, thereby reducing the over-fitting in the data and improving the accuracy of the models. The models, which we term as QSPRs, are in the form of equations with the target properties calculated as a linear combination of the reduced descriptor base. This equation can then be rapidly applied to new material and processing conditions, to significantly expand the material knowledge base, while being based on the governing physics and therefore relevant to development of virtual databases. This design strategy proposed as a standardized method for development of virtual materials databases is shown in figure 1. In this review, we utilize RST for defining the critical descriptors while considering uncertainty and PLS for building predictive models linking the reduced descriptor set with materials properties, while capturing the physics of the target properties and addressing the multi-collinearity in the data.

The starting data for inorganic scintillators integrates both experimental and computational data. An issue with the data is the relatively small data sizes and the sparseness of the data. This represents a challenge by introducing uncertainty into the design process. We address that in this work through the application of rough sets and also by developing a physics-guided design strategy. In this work, we consider among other component characteristic specific attributes of scintillator compounds, other descriptors such as density of the host crystal, effective atomic number, absorption length, Stoke’s shift (S), valence electron (VE) factor, size (SZ) factor, electrochemical (EC) factor, photoabsorption coefficient, absorption length, and crystal field depression. The details of these descriptors are provided in the references [19–22]. Our objective is to reduce the descriptor space to only those descriptors correlated with LY.

## 3. Data classification via RST

One of the challenges in materials informatics is identifying the minimum amount of information to describe a problem, in this case the minimum amount of information to physically capture the relationship between descriptors and property. When considering large descriptor-bases, we need to address challenges associated with large amounts of data, identify correlations amongst descriptors which we can correlate to the driving physics, and identify which descriptors to input into the models for developing QSPRs. This work then reduces the amount of descriptors required to be calculated to reduce the data requirements to a minimum. We have previously applied principal component analysis (PCA) to identify the minimum number of required descriptors for a variety of problems [23–28]. In PCA, the input descriptor matrix is converted into two matrices: the scores and loadings matrices. The scores matrix describes the conditions, such as chemistry and processing, while the loadings matrix describes the responses (i.e. descriptors and properties) [29–33]. The loadings matrix defines the correlation between properties and material descriptors. Two properties which have the same PC values provide redundant information. We therefore use these relationships to differentiate between necessary data descriptions and those which do not provide unique information, thereby reducing the data calculation requirements. While we do not use that method in this review, the mathematics used in PLS for regressions builds on the mathematics of PCA. We instead utilize RST to identify the descriptors most correlated with LY and define the ranges of those descriptors associated with high LY. RST is based on dividing the parameter space with multiple cuts which differentiate between the property groupings. The number of cuts added when including an additional descriptor correlates with the importance of that descriptor on the target property. Rough sets differ from PCA, which instead provides a projection of the data from which correlation between descriptor and property can then be calculated. Rough sets add an uncertainty measure by defining that a compound belongs to a set, but also includes the option that a compound possibly belongs to a set.

To describe the logic of RST, we describe the analysis of density and EC factor as the material descriptors with LY as the property. The decision space should be a categorical response which is a mandatory condition to start with rough set analysis. Therefore the response, in this case the LY, is classified into four different categories (low, medium, high and very high). The cutoffs for each category are as follows: low LY from 0 to 10 000; medium LY from 10 000 to 20 000; high LY from 20 000 to 30 000; and very high LY is greater than 30 000.

**Figure 2.**
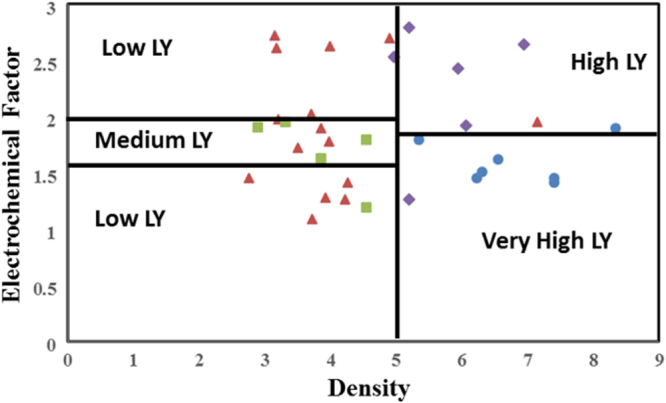
Subset *B* in the example of rough set approach for defining separation of light yield categories. Low light yield compounds are shown in red triangles, medium light yield compounds are green squares, purple diamonds are high light yield compounds, and the very high compounds are shown blue circles. In this figure, we show three cuts (shown as lines dividing the classes of material). Two of the cuts are for density and one cut is for Stoke’s shift. The accuracy of these cuts in discriminating the light yield categories is not sufficient, which indicates the need for further descriptors to be added.

Rough set builds on traditional set theory by defining a boundary region. Within the lower approximation region, a compound belongs to a specific class (in this case LY category), outside the outer approximation, the compound does not belong to that class. However, in the boundary region, defined as the space between the upper and lower approximations, a compound may possibly belong to the class. We first define an information system *S* as *S* = (*U*, *A*), where *U* are the components of the system, in this case scintillator chemistries, and *A* are the attributes of the system, in this case the different material descriptors and properties. We also define the subset of *A*, which we term as *B*. *B* therefore contains a portion of the attributes contained within *A*. In the example following, *B* = (density, EC factor). *B* for the scintillator case is shown in figure 2, where the categories of scintillators are discriminated. If we consider only these two descriptors, we can classify the LY classes with only four cuts. However, 28% of the compounds are misclassified in this case. We therefore need to include further parameters to improve the discrimination of the compounds. This process of parameter combinations and number of cuts is performed for every permutation to identify the best combination of accuracy of set approximation and quality of classification, which is based on an uncertainty metric.

**Figure 3.**
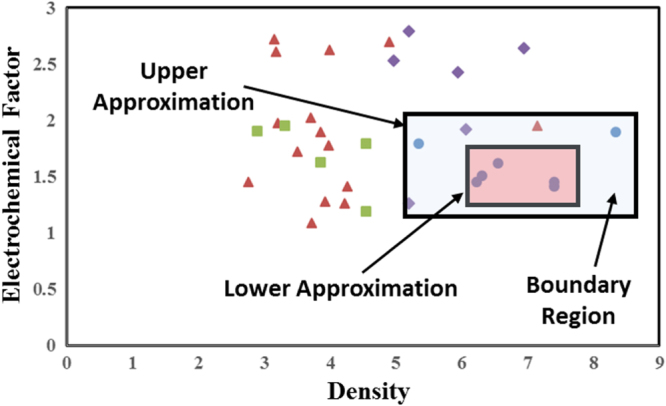
Definition of lower approximation, upper approximation, and boundary region for very high light yield compounds is shown. The lower approximation is defined as the region where every compound is within the same class, in this case very high light yield. The upper approximation is then the region which contains that class while also some compounds in other classes. The boundary region is defined as the region in between the lower and upper approximation. By introducing this boundary region, where compounds may possibly belong to a class, we introduce uncertainty into the analysis.

We define two further parameters: the lower approximation 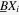 and the upper approximation 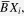 where *X* is a subset of *U*. Effectively, we can consider this as defining the range of descriptor values where every entry belongs to a specific class/category and the range which contains all the values of a class/category. In this way, every entry within the lower approximation material belongs to the same class, while in the upper approximation, all materials of a class are contained, while at the same time materials of other classes may also be present. In a situation where all compounds of a class were clustered together in the attribute space, the lower and upper approximations would be equal. The definition of the boundary region for low LY compounds within *B* = (density, EC factor) for very high LY compounds is provided in figure 3.

To determine the appropriate number of cuts and the appropriate parameters to consider, we define two parameters: accuracy of set approximation and quality of classification (equations (1) and (2)). These provide ratios of those compounds which belong to a set versus those that may belong to a set and is used to define uncertainty and accuracy of the rough set. These metrics assess the importance of individual attributes in determining the decision class. The accuracy of the set approximation is the ratio of the compounds which are within the lower approximation to those within the upper approximation, and therefore is correlated with the percent of compounds which may possibly belong to a class. The quality of classification is defined as the number of compounds within the lower approximation divided by the entire number of compounds in the information system.

1
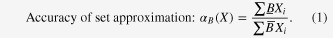


2
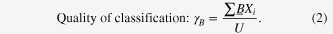


**Figure 4.**
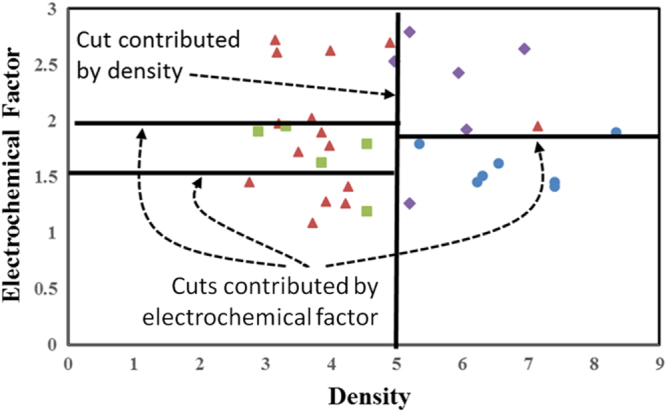
The approach for defining relative importance of a descriptor on a property. The relative importance is defined as the number of cuts associated with a descriptor versus the total number of cuts. In this example, four cuts are shown, with one associated with density and three with electrochemical factor. We therefore identify electrochemical factor as approximately three times more important than density in this example.

Every combination of parameters and cuts is used and the accuracy and quality of the models are determined. The selection of model is then based on that which provides the best combination of these characteristics. From the final model selected, we identify the descriptors which are most important for the target property and the relative importance of that descriptor. The importance of the descriptor is defined as the number of cuts contributed by the descriptor versus total number of cuts. For example, density adds one cut in our example, while EC factor adds three cut (figure 4). In this example, density is therefore defined as having 25% relative importance on LY while EC factor has 75% relative importance. Further, the critical descriptor ranges are defined as the values where the lower approximations for a category occur. In this case, the critical values for density to achieve high LY are between six and eight, and EC factor from 1.25 to 1.75. When adding more parameters and thus cuts, these values can become smaller ranges. This defines then the measurement of the relative importance of a descriptor on a property and the critical design ranges for designing materials for target property.

This approach operates as a descriptor reduction by identifying redundant descriptors. That is, if the addition of a descriptor does not provide classification improvement while reducing the number of cuts, then the descriptor provides no relevant information for the property of information and can then be considered redundant. This descriptor is then removed in what is terms a reduct. The analogous approach is followed for the number of cuts. The cuts are removed if they do not provide relevant information. This reduction of cuts is referred to as discretization. The combination of these two aspects then identifies the minimum amount of information required for defining a property category, and is termed as a disreduct. The outputs shown in the results section are based on defining this disreduct, where the number of descriptors and cuts are minimized without reducing accuracy and quality of the classifications.

## 4. Development of QSPRs

In PLS the training data is converted to a data matrix with orthogonalized axes, which are based on capturing the maximum amount of information in fewer dimensions. The relationships discovered in the training data can be applied to a test dataset based on a projection of the data onto a high-dimensional hyperplane within the orthogonalized axis-system. Typical linear regression models do not properly account for the co-linearity between the descriptors, and as a result the isolated impact of each descriptor on the property cannot be accurately known. However, by projecting the data onto a high-dimensional space defined by orthogonal axes which are comprised of a linear combination of the descriptors defining the properties, the impact of the descriptor on the property can be identified independent of all other descriptors.

**Figure 5.**
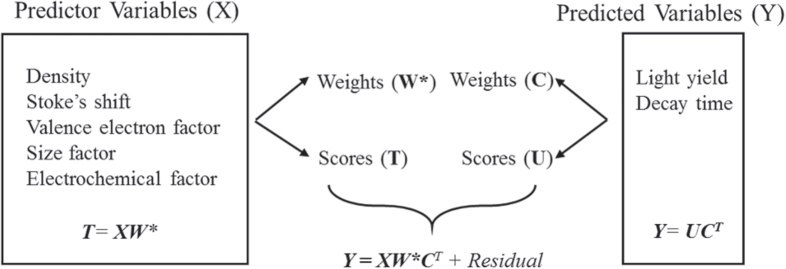
The description of data used in this analysis and the logic for developing a QSPR. The regression coefficients are defined as **W**∗**C**^T^, which is the product of weights for converting predictor and predicted variables into latent variable space, respectively. PLS operates by performing separate PCA-like analyses on the predictor matrix and the predicted matrix, outputting the weights needed to convert the matrix to latent variable space and the values of the materials in latent variable space (scores). The PLS mathematics perform matrix transformations so that the final predictive model calculates the properties as a function of the reduced descriptor set, the weights (importance) of the individual descriptors, and the weights of the predicted variables for the training data. This results in a computationally efficient model for rapid prediction of properties.

In PLS the training data is converted to a data matrix with orthogonalized axes, which are based on capturing the maximum amount of information in fewer dimensions, and thus building on the mathematics of PCA [34–38]. The relationships discovered in the training data can be applied to a test dataset based on a projection of the data onto a high-dimensional hyperplane within the orthogonalized axis-system. With PLS, the properties of the training data are modeled as a function of the controllable parameters such as chemistry and processing. Typical linear regression models do not properly account for the co-linearity between the descriptors, and as a result the isolated impact of each descriptor on the property cannot be accurately known. However, by projecting the data onto a high-dimensional space defined by axes which are comprised of a linear combination of the composite descriptors and also orthogonalized, the impact of the descriptor on the property can be identified independent of all other descriptors. Therefore, PLS is used here to identify structure–property relationships, with the logic of PLS is provided in figure 5.

The PLS prediction requires two input matrices: a matrix which contains descriptors related to the input conditions and a matrix containing the values which are to be predicted, building a model between the input descriptors and the descriptor to be predicted. The predictor descriptors are the reduced descriptor set which we identified in the rough set analysis. The predicted descriptors are the properties we want to predict. In this case, the predicted variable matrix contains LY and decay time. PLS operates by performing separate PCA-like analyses on the predictor matrix and the predicted matrix, outputting the weights needed to convert the matrix to latent variable space and the values of the scintillator compounds in latent variable space (scores). The scores plot in both cases has dimensions of compounds versus principal components (latent variables). Therefore, the dimensions of both the predictor scores matrix and the predicted scores matrix are the same. To link the predictor and prediction matrices, we substitute the predicted scores matrix for the predictor scores matrix, so the input data is defined as the relationships in the reduced descriptor set and the relationship of the compounds in terms of properties. This approach allows us then to integrate the variance in the various levels of data descriptions.

To ensure accuracy of the QSPR modeling and to verify that we are not over-fitting the data, we employ a cross validation to the predicted results. To this end, we compute both the root mean square error of calibration (RMSEC) and the root mean square error of cross validation (RMSECV). We perform a leave-one-out (LOO) cross validation and measure the accuracy of the model with and without the variable left out in the LOO approach. This step is repeated for removing each sample from the training data. That is, a model is built removing each sample, thereby ensuring that the physics captured in the model development is sufficiently robust that it can be used on new materials. The RMSEC and RMSECV values are then used to define the final predictive model. To select the number of latent variables with a suitable combination of accuracy and robustness, we define a criteria for selection of latent variables based on the ratio of RMSECV (*m*)*/*RMSECV (*m* + 1), where *m* is equal to the number of latent variables. From our criteria, *m* is selected such that it is the maximum number with the ratio below the threshold value of unity.

## 5. Case study: database for multifunctional properties

In order to calculate the impact of a descriptor on property, specifically on LY, the disreduct set was determined and the ratio of cuts contributed by each reduced descriptor was calculated. Further, based on the location of the cuts, the range for high LY compounds in terms of descriptor values was identified. The descriptor base was reduced to five descriptors which were identified as providing relevant information in the classification of compounds into the LY categories. The five descriptors identified as relevant for predicting LY were density, S shift, VE factor, SZ factor , EC factor. This therefore identifies the input into the PLS model. As this represents a number significantly less than the number of compounds in the training data, this provides an appropriate input in terms of both robustness and accuracy. By correlating the ratio of cuts for each descriptor, we find that S shift is the most important descriptor, followed by VE factor, SZ factor, and EC factor, and finally with density having a minor impact. The other descriptors have been removed as they were identified as not impacting the LY of the compounds.

The relative importance of each descriptor on the LY is as follows: density—5%, S shift-33%, VE factor—20%, SZ factor—20%, and EC factor—23%. The ranges of values for high LY, as defined by corresponding to the high LY boundary provide guidelines for designing new materials. Density values of seven to eight, S shift values from 1000 to 2500, VE factor of four to five, SZ factor from 1.75 to two, and EC factor from 1.25 to 1.45 correspond to the highest LY regions. This approach has therefore provided both a reduced descriptor set improving prediction accuracies and reducing data creation requirements, while also providing design rules for selection of compounds. The application of rough sets has defined the input into the PLS regression. By incorporating uncertainty, the accuracy, tractability and particularly the robustness of the regressions is enhanced.

**Figure 6.**
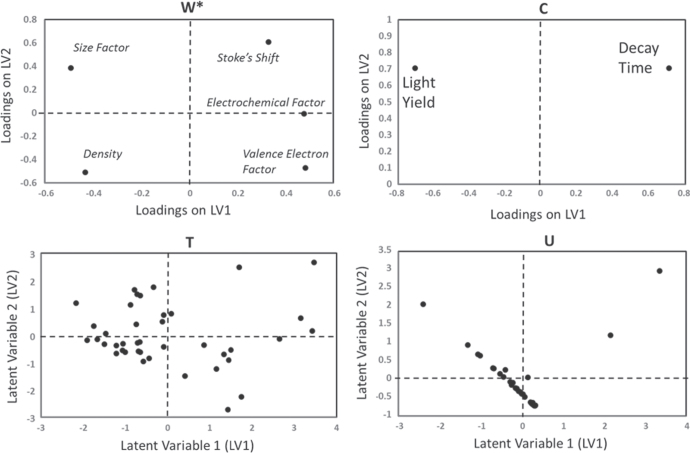
The data matrices for the PLS regressions, based on the reduced descriptor set from the rough set theory. By minimizing the descriptor base (**X**), we improve the modeling of the predictor variables (**Y**), which for our case includes light yield and decay time. PLS operates by performing PCA analyses on each matrix, and to then combine the matrices as defined in figure 5. This combination allows for co-linearity of variables to be defined so that over-fitting of properties is minimized.

**Figure 7.**
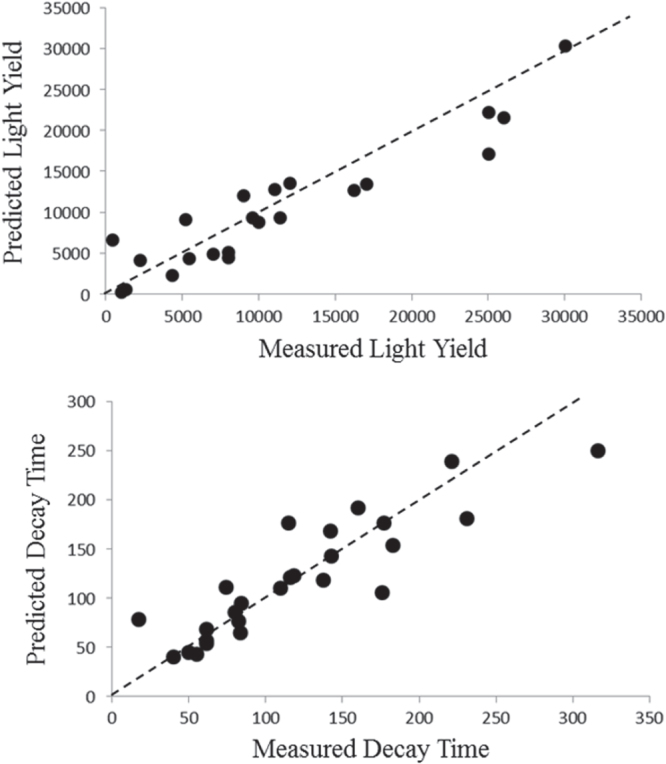
In order to screen the endless number of possible scintillator host lattice chemistries, we developed QSPRs for light yield and decay time as a function of the reduced descriptor set identified through the rough set analysis. The accuracy of these predictions allows us to rapidly screen a very large search space to identify those compounds with meet our design requirements of high light yield and fast decay time.

We have reduced the massive descriptor search space to five descriptors, which we use to predict two properties (LY and decay time). These five descriptors and two properties provide data matrices input into the PLS regression (figure 6). The combination of the different regressions thus provides models which are used to predict very rapidly the LY and decay time of new compounds. The accuracy of both regression models provides a significant acceleration which incorporates physics, uncertainty and empirical measurements (figure 7). This work then is used to accelerate the data calculation and provides a significant library for discovering scintillating materials.

**Figure 8.**
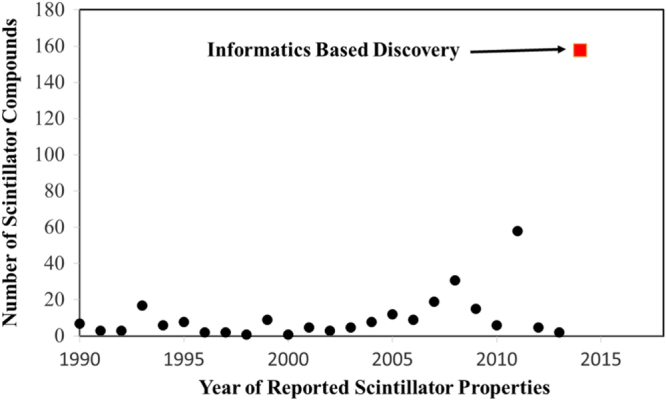
Acceleration of scintillator discovery. Scintillator discoveries are defined as those which meet meet minimal requirements for light yield and decay time employing our QSPR, which also addresses issues in uncertainty. The prior discoveries in scintillator compounds are taken from reference [39] and the references therein. This demonstrates the applicability of our approach for significantly accelerating the discovery of new materials, while reducing the limitations and assumptions of other approaches. This work provides a generalized framework for developing large ‘virtual’ material libraries.

Having developed a QSPR, we can then apply it to a ‘virtual’ library of compounds. While the compounds can theoretically contain any combination of elements, only those represented within the training data provide sufficiently high confidence in predictions. For example, if elements A, B and C are represented within the training data, then any compound containing a permutation of A, B and C may be included in the predictions. In this way, a significant number of compounds are rapidly calculated and those that have the target properties can be identified. Through the development of the QSPR which is based on descriptors capturing uncertainty and being physically relevant, we have significantly accelerated the process of data creation (figure 8).

## 6. Summary

In this review, we have outlined a new framework for accelerated development of materials databases with targeted multifunctional properties. Traditional database development is dependent upon the parameterization available for different types of computation or the limitation of experiments. Here we show that the use of informatics methods can permit the establishment of multi-objective data classifications and rule based design and prediction of structure–property relationships. This now creates a new paradigm in the construction, organization and utilization of databases. Informatics now can transform the traditional role of databases as repositories of known information into ‘data laboratories’ for generating new knowledge.
